# Earliest Palaeocene purgatoriids and the initial radiation of stem primates

**DOI:** 10.1098/rsos.210050

**Published:** 2021-02-24

**Authors:** Gregory P. Wilson Mantilla, Stephen G. B. Chester, William A. Clemens, Jason R. Moore, Courtney J. Sprain, Brody T. Hovatter, William S. Mitchell, Wade W. Mans, Roland Mundil, Paul R. Renne

**Affiliations:** ^1^ Department of Biology, University of Washington, Seattle, WA 98195, USA; ^2^ Department of Paleontology, Burke Museum of Natural History and Culture, University of Washington, Seattle, WA 98195, USA; ^3^ Department of Earth and Space Sciences, University of Washington, Seattle, WA 98195, USA; ^4^ Department of Anthropology, Brooklyn College, City University of New York, 2900 Bedford Avenue, Brooklyn, NY 11210, USA; ^5^ Department of Anthropology, The Graduate Center, City University of New York, 365 Fifth Avenue, New York, NY 10016, USA; ^6^ New York Consortium in Evolutionary Primatology, New York, NY, USA; ^7^ Department of Integrative Biology, University of California, Berkeley, CA 94720, USA; ^8^ Museum of Paleontology, University of California, Berkeley, CA 94720, USA; ^9^ Department of Earth and Planetary Science, University of California, Berkeley, CA 94720, USA; ^10^ Honors College, University of New Mexico, Albuquerque, NM 87131, USA; ^11^ Department of Earth and Planetary Sciences, University of New Mexico, Albuquerque, NM 87131, USA; ^12^ Department of Geological Sciences, University of Florida, Gainesville, FL 32611, USA; ^13^ Minnesota IT Services, St Paul, MN 55155, USA; ^14^ Berkeley Geochronology Center, 2455 Ridge Road, Berkeley, CA 94720, USA

**Keywords:** primates, Purgatoriidae, plesiadapiforms, Cretaceous–Palaeogene boundary, frugivory

## Abstract

Plesiadapiform mammals, as stem primates, are key to understanding the evolutionary and ecological origins of Pan-Primates and Euarchonta. The Purgatoriidae, as the geologically oldest and most primitive known plesiadapiforms and one of the oldest known placental groups, are also central to the evolutionary radiation of placentals and the Cretaceous-Palaeogene biotic recovery on land. Here, we report new dental fossils of *Purgatorius* from early Palaeocene (early Puercan) age deposits in northeastern Montana that represent the earliest dated occurrences of plesiadapiforms. We constrain the age of these earliest purgatoriids to magnetochron C29R and most likely to within 105–139 thousand years post-K/Pg boundary. Given the occurrence of at least two species, *Purgatorius janisae* and a new species, at the locality, we provide the strongest support to date that purgatoriids and, by extension, Pan-Primates, Euarchonta and Placentalia probably originated by the Late Cretaceous. Within 1 million years of their arrival in northeastern Montana, plesiadapiforms outstripped archaic ungulates in numerical abundance and dominated the arboreal omnivore–frugivore niche in mammalian local faunas.

## 1. Introduction

Plesiadapiforms are crucial to understanding the evolutionary and ecological origins of primates and other euarchontans (treeshrews and colugos) as well as the traits that separate those groups from other mammals [1]. Plesiadapiforms are a paraphyletic assemblage of 11 extinct families and more than 150 species from the Palaeocene and Eocene of North America, Europe and Asia (see [2] for most up-to-date species list). Researchers have debated the systematic affinities of this group, with some suggesting that some or all plesiadapiform taxa are more closely related to other euarchontans (e.g. dermopterans) than they are to primates, and others interpreting plesiadapiforms as stem primates (see [2] for a review). Recent phylogenetic analyses, which have benefited from newly discovered, exceptionally complete fossils, support the latter hypothesis and consistently place plesiadapiforms as successive sister-taxa to Primates (e.g. [3,4]; but see [5]). Here we use Pan-Primates [6] to designate the total clade that includes the crown clade (Primates [7]) and all stem primates (including plesiadapiforms). The Purgatoriidae, as the oldest and most primitive known plesiadapiform family [8–10], are particularly important to understanding primate ancestry. Researchers can use their morphology to inform character-state polarity in phylogenetic analyses, resolve broader relationships among stem and crown primates, and characterize the ecology of the earliest members of Pan-Primates. Purgatoriids were also among the first known placental mammals to diversify, both taxonomically and ecologically, following the Cretaceous–Palaeogene (K/Pg) mass extinction that resulted in the loss of all non-avian dinosaurs [11,12]. Understanding the detailed pattern of this seminal diversification event thus has implications for understanding the evolutionary radiation of placentals and the K/Pg biotic recovery on land.

The Purgatoriidae comprise two genera, *Purgatorius* and *Ursolestes*, and seven named species [2]. Van Valen and Sloan [13] named two species, both from northeastern Montana: the type species, *P. unio*, on the basis of six isolated teeth from the early Palaeocene (Pu3 subinterval of the Puercan North American land mammal ‘age’) Purgatory Hill locality, and *P. ceratops* from the Harbicht Hill locality, then considered latest Cretaceous in age. Revised stratigraphic interpretations at Harbicht Hill suggest that the fossil assemblage is a mixture of both earliest Palaeocene and reworked latest Cretaceous taxa [14,15]. In addition to its age uncertainties, *P. ceratops* is known by only a single, poorly preserved lower molar that most authors consider non-diagnostic (e.g. [16,17]). The first large sample of isolated teeth and dentigerous dentary fragments of *Purgatorius* were reported from the Pu3 Garbani Channel localities in northeastern Montana [18]. Initially referred to *P. unio*, Van Valen [19] later placed them in a new taxon, *P*. *janisae*. Buckley [20] also described a large sample of teeth from the Pu3 Simpson Quarry in southcentral Montana, for which he named *P. titusi*, although we provisionally follow Silcox [21] in considering *P. titusi* a junior synonym of *P. unio*. The only other known purgatoriid genus, *Ursolestes,* is also from Simpson Quarry and includes but a single species, *U. perpetior*, that has dental dimensions more than twice as large as those of all species of *Purgatorius* [22]. Two other plesiadapiform species, both members of *Pandemonium* (Family *incertae sedis*), also occur in the Puercan: *Pandemonium* *dis* from the Pu3 Purgatory Hill locality of northeastern Montana [19] and *Pandemonium hibernalis* from the ?Pu2 Schowalter locality of southern Alberta [23]. In the light of the uncertainties about *Purgatorius ceratops*, the earliest purgatoriid (and plesiadapiform) known to date is *P*. *coracis*, erected on the basis of 29 isolated teeth and two fragmentary jaws from the Medicine Hat Brick and Tile Quarry, Rav W-1 horizon, from southwestern Saskatchewan [24]. That locality occurs in magnetochron C29R but preserves other mammals thought to be of Pu2-aspect. Another taxon, *P. pinecreeensis*, was reported from the Pine Cree Park locality near Rav W-1, but that locality is more questionably considered Pu2 in age [25].

**Figure 1.**
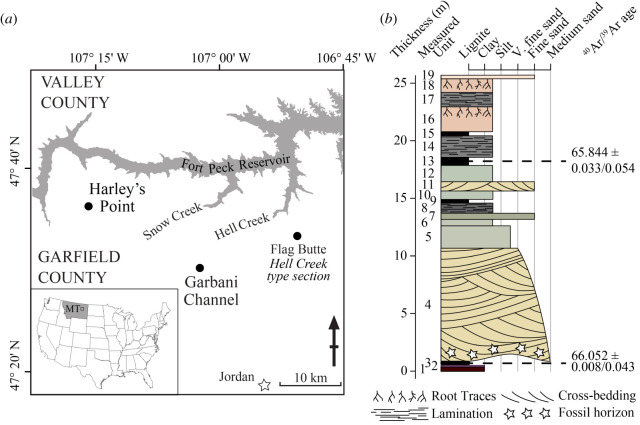
(*a*) Location map showing the study area in northeastern Montana, USA. Fort Peck Reservoir is shown in grey. Circles show locations of localities discussed here including Harley's Point and Garbani Channel. The location of the Hell Creek type section, Flag Butte, is also shown. (*b*) Stratigraphy of a composite section compiled from the most proximal outcrops to Harley's Point locality (shown in electronic supplementary material, figure S1b). Ages shown were determined in [26], for details see the electronic supplementary material.

Here, we report on the discovery of even earlier occurrences of *Purgatorius*. Five isolated teeth were recovered from the early Palaeocene Harley's Point locality in the lowermost part of the Tullock Member of the Fort Union Formation in northeastern Montana (figure 1). Two lower molars from this sample are referred to *P. janisae*, which is otherwise only known from stratigraphically higher deposits in the Tullock Member (i.e. Garbani Channel localities); another lower molar is morphologically distinct from all known purgatoriid species, leading us to erect a new taxon; and two upper molars are tentatively referred to that new taxon. During our comparative study, we encountered three dentigerous dentary fragments from the Pu3 Garbani Channel assemblage that also represent the new taxon. With new stratigraphic, biochronological, sedimentological and geochronological data from the Harley's Point locality and surrounding outcrop (see the electronic supplementary material), we constrain the age of the earliest purgatoriids to the early Puercan (Pu1), to magnetochron C29R, and to within 208 thousand years (kyr) after the K/Pg boundary (KPB), with a likelihood that their age could be as little as 105–139 kyr post-KPB. Note that *Purgatorius coracis* from southwestern Saskatchewan also occurs in C29R, but the associated Pu2-aspect mammalian fauna suggests that it is younger than the Pu1 specimens described herein. ­Thus, the occurrence of at least two species of *Purgatorius* at the Pu1 Harley's Point locality provides the strongest support to date that purgatoriids and, by extension, Pan-Primates, Euarchonta and Placentalia must have originated in the Late Cretaceous [24].

### 1.1. Institutional abbreviations and conventions

LACM, Los Angeles County Museum, Los Angeles, California, USA; UCMP, University of California Museum of Paleontology, Berkeley, California, USA; UWBM, University of Washington Burke Museum, Seattle, Washington, USA. We use standard convention in referring to lower dentition with lower-case letters (p, premolar and m, molar) and upper dentition with upper-case letters (P and M, respectively). The number following the letter designates tooth position in the series.

## 2. Results

### 2.1. Systematic palaeontology

The fossil specimens described herein are permanently stored in the collections of the UCMP. All digital models are available on MorphoSource (https://www.morphosource.org/).

Primates Linnaeus, 1758; Purgatoriidae Gunnell, 1989; *Purgatorius* Van Valen and Sloan, 1965.

***Purgatorius janisae*** Van Valen, 1994

**Figure 2.**
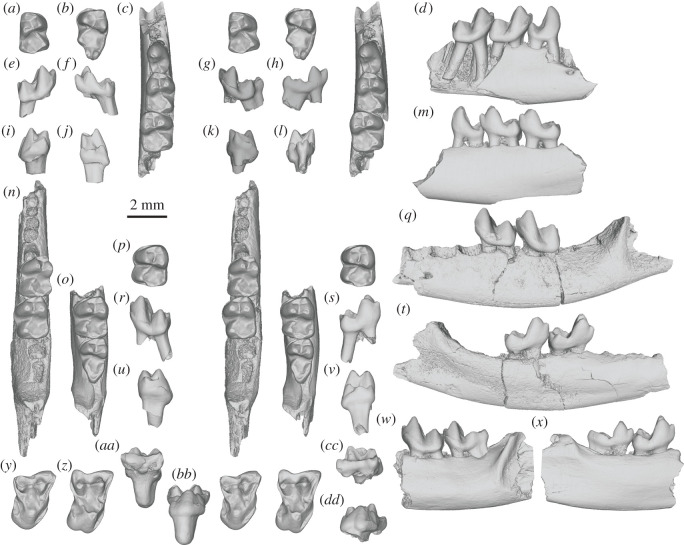
*Purgatorius* from Harley's Point (Pu1) and Garbani Channel (Pu3) localities of northeastern Montana, USA. Images are three-dimensional surface renderings derived from µCT scans: *P. janisae* in stereo occlusal, buccal, lingual, mesial and distal views (*a,e,f,i,j*) UCMP 150018, right m1 and (*b,g,h,k,l*) UCMP 192398, left m3; *P. mckeeveri* sp. nov. in stereo occlusal, buccal and lingual views (*c,d,m*) UCMP 157977 (holotype), incomplete right dentary with p4–m2, (*n,q,t*) UCMP 111586, incomplete left dentary with m1–2, (*o,w,x*) UCMP 189505, incomplete left dentary with m2–3 and in stereo occlusal, buccal, lingual, mesial and distal views (*p,r,s,u,v*) UCMP 150021, left m2; and *Purgatorius* cf. *P. mckeeveri* in stereo occlusal, buccal and lingual views (*y,aa,bb*) UCMP 150019, right M2, and (*z,cc,dd*) UCMP 150020, right M2.

Referred specimens. UCMP 150018, right m1, and UCMP 192398, left m3 (figure 2*a,b,e–l*).

Horizon locality. The early Puercan (Pu1) ‘Harley's Point’ UCMP locality V77087 from the lowermost Palaeocene Tullock Member, Fort Union Formation, Garfield County, Montana, USA.

Description. See the electronic supplementary material.

***Purgatorius mckeeveri*** sp. nov.

Etymology. ‘*McKeever*’ honours Frank McKeever, who was among the first residents of Garfield County to facilitate the fieldwork of Harley J. Garbani in 1965, and the family of John and Cathy McKeever, who have since supported our fieldwork at the Harley's Point locality, where the oldest specimen of this new taxon was recovered.

Holotype. UCMP 157977, right dentary fragment with p4–m2 (figure 2*c,d,m*).

Hypodigm. UCMP 111586, left dentary fragment with m1–2, UCMP 189505, left dentary fragment with m2–m3, and UCMP 150021, left m2 (figure 2*n–x*).

Horizon localities. The ‘Garbani Channel-NW Harley's High’ UCMP locality V73080 (holotype and UCMP 111586) and the ‘Garbani Channel-NW Main Quarry’ UCMP locality V73082 (UCMP 189505), both from late Puercan (Pu3), and the ‘Harley's Point’ UCMP locality V77087 (UCMP 150021) from early Puercan (Pu1), all from the lowermost Palaeocene Tullock Member, Fort Union Formation, Garfield County, Montana, USA.

Diagnosis. Differs from all other purgatoriids in having lower molars with more inflated cusps, rounded crests, and a paraconid that is higher on the trigonid and more appressed to the metaconid (especially on m2). Further differs from *P. pinecreeensis* and *P. ceratops* in having slightly larger lower molar dimensions. Further differs from *P. janisae*, *P. unio* and *P. coracis* in having a paraconid that is distinct from the paracristid and a shallower protocristid notch (on m1 and m2) with closer proximity of the metaconid and protoconid apices. Further differs from *P. janisae*, *P. unio*, *P. ceratops* and *P. pinecreeensis* in having a J-shaped (not L-shaped) paracristid on m2. Further differs from *P. janisae* and *P. pinecreeensis* in having a m3 that is narrower (particularly the talonid) relative to m2 with smaller talonid cusps and a shallower talonid basin. Further differs from *P. unio* and *P. coracis* in having lower molars with a larger paraconid and a more transversely oblique postvallid. Further differs from *P. janisae* in having a p4 with a lower protoconid and poorly developed talonid and without a paraconid, in having lower molars with a smaller, not mesially projecting paraconid, and a less transversely oblique postvallid, and in having a lower p4:m1 length ratio and a lower trigonid:talonid length ratio on m1 and m2. Further differs from *P. coracis* in having lower molars with a paraconid that is more lingual in position and a shallower talonid basin. Further differs from *P. unio* in lacking a p4 paraconid (variably present in *P. unio*), having a p4 talonid with a less distinct lingual cusp (entoconid) and having molars with smaller, less distinct talonid cusps and a higher trigonid:talonid length ratio on m1 and m2.

Description. See the electronic supplementary material.

***Purgatorius*** cf. ***P. mckeeveri***

Referred specimens. UCMP 150019, right M2, and UCMP 150020, right M2 (figure 2*y–dd*).

Horizon locality. The early Puercan (Pu1) ‘Harley's Point’ UCMP locality V77087 from the lowermost Palaeocene Tullock Member, Fort Union Formation, Garfield County, Montana, USA.

Description. See the electronic supplementary material.

### 2.2. Comparisons

On the basis of dental morphological comparisons with a broad range of latest Cretaceous (Lancian) and early Palaeocene (Puercan and Torrejonian) mammals, we conclude that these specimens are most similar to those of purgatoriid plesiadapiforms, specifically to species of *Purgatorius*.

The two lower molars (UCMP 150018 and UCMP 192398) from the Harley's Point locality that we refer to *P*. *janisae* are almost identical to those of the holotype (UCMP 107406 [18,19]). The incipient double hypoconulid on the m3 (UCMP 192398; figure 2*b,l*) is not developed on the holotype but occurs on some m3s in the Garbani Channel sample of *P*. *janisae* [18].

The new species, *Purgatorius mckeeveri*, from the Harley's Point and Garbani localities is distinguishable from *Ursolestes perpetior* notably in its smaller size but also in dental morphology (e.g. more developed p4 talonid basin [22]). It is most similar in size to *P. janisae* and *P. unio* (slightly larger than *P. coracis*, *P. pinecreeensis* and *P. ceratops*). It is morphologically distinct from all of those taxa in having lower molars with (i) a relatively taller molar trigonid (trigonid:talonid height ratio on m1 and m2) with (ii) more rounded crests, (iii) more inflated cusps, and (iv) a paraconid (especially on m2) that is higher on the trigonid and more appressed to the metaconid (no distinct valley separating them). Additional comparisons with each *Purgatorius* species can be found in the electronic supplementary material.

The Harley's Point upper molar specimens (UCMP 150019 and 150020) are morphologically distinct from those of all other purgatoriid species. They are notably smaller than the M2 of *Ursolestes perpetior* [22], larger than the M1 of *Purgatorius pinecreeensis*, and similar in size to the upper molars of all other purgatoriids*.* They are more transverse than M2s referred to *P*. *janisae* but not as transverse as those of *P. unio*, *P. coracis, U. perpetior* and the M1 of *P. pinecreeensis*. The protocone in both UCMP 150019 and 150020 is not as mesiodistally compressed as in *P. unio* and *P. coracis* and the M1 of *P. pinecreeensis*; and it is in a more lingual position on the crown compared with that of *P. unio*. The Harley's Point specimens differ from M2s of *P. janisae* in that the buccal margin of the crown is asymmetrical (i.e. divided into unequal lobes by the ectoflexus), the metacone is slightly more buccal on the crown, the protocone apex is slightly more buccal on the crown (slightly less so in UCMP 150020), and the protocone base is more lingually expanded (but not as much as in *P. unio*); in turn, the lingual part of the crown is more twisted [19,21] than in *P. janisae* (but less than so than in *P. unio*). UCMP 150020 has a faint furrow (or cleft) that extends ventrally from the lingual base of the protocone and fades away midway to the apex (figure 2*z,dd*). This furrow creates a slightly bilobed outline of the lingual margin in occlusal view that is unique among known purgatoriid species but reminiscent of the more pronounced bilobed condition of some palaechthonid and paromomyid plesiadapiforms, such as *Paromomys farrandi* [27]. Thus, despite a number of features shared with the upper molars of known purgatoriid species, these specimens are not attributable to any taxon that is known from upper molars. Because lower molars of *P. mckeeveri* occur at this locality, we tentatively assigned these specimens to the new taxon. The coronal dimensions and occlusal morphology of the upper molars are consistent with this hypothesis.

### 2.3. Taxonomic diversity of early Palaeocene plesiadapiforms versus archaic ungulates

**Figure 3.**
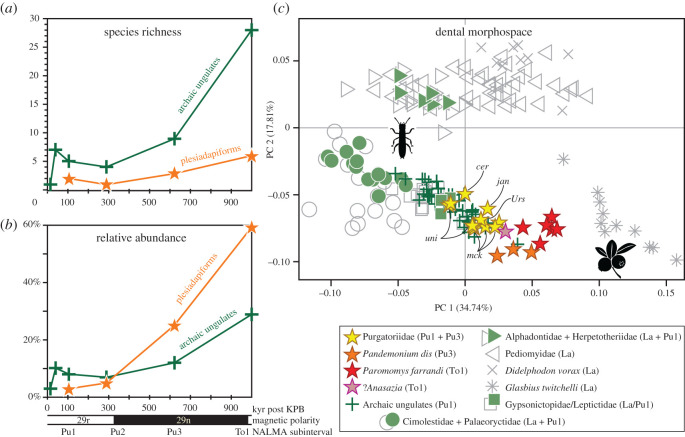
Taxonomic diversity and dental morphospace of early Palaeocene plesiadapiforms versus archaic ungulates. (*a*) Species richness and (*b*) relative abundances of plesiadapiforms (stars) and archaic ungulates (plus signs) in mammalian local faunas from northeastern Montana, USA. Data are from [12,28,30] and are provided in the electronic supplementary material. (*c*) PC1 versus PC2 plot of two-dimensional geometric morphometric analysis of lower molars illustrating dental morphospace occupancy of Lancian and early Puercan (Pu1) therians from northeastern Montana with additional plesiadapiforms from later in the Puercan (Pu3) and the early Torrejonian (To1) NALMA subintervals. *Purgatorius ceratops* (cer) = ?Pu1; *P. janisae* (jan) and *P. mckeeveri* (mck) = Pu1 and Pu3; and *P. unio* (uni) and *Ursolestes perpetior* (Urs) = Pu3. Data are from [31] and this study. The specimen list is provided in the electronic supplementary material.

Plesiadapiforms are absent from the oldest local faunas of the Palaeocene of northeastern Montana (the Z-Line local fauna [28] and the Worm Coulee 1 local fauna [12,29], from *ca* 25 and 80 kyr post-KPB, respectively; figure 3*a,b*). Their absence is probably not an artefact of sampling, given that the Worm Coulee 1 local fauna is represented by greater than 900 specimens of many other small-bodied mammals [12]. Plesiadapiforms first appear in the Harley's Point local fauna and the slightly younger Coke's Clemmys local fauna (*ca* 250 to 328 kyr post-KPB [28]), represented by up to three *Purgatorius* spp. and less than 5% of all mammalian individuals (figure 3*b*). In the younger Garbani Channel local fauna (*ca* 311 to 934 kyr post-KPB, with a likely age between 584 and 691 kyr post-KPB, see electronic supplementary material), plesiadapiforms are represented by slightly more species [12,13,19] and a much greater relative abundance (25%). Although the relative abundance of purgatoriids is much lower in the Horsethief Canyon (*ca* 855 to 1.148 Myr post-KPB [32]) and Farrand local faunas (*ca* 934 kyr to 1.01 Myr post-KPB), the paromomyid plesiadapiform *Paromomys farrandi* makes up approximately 56% of all individuals [27,30]. In comparison, archaic ungulates occur earlier and are represented by many more species (seven species in the Worm Coulee 1 local fauna and 28 species in the Horsethief Canyon and Farrand local faunas) but have fairly low relative abundance (less than or equal to 12%) until *ca* 1 Myr post-KPB (29%).

### 2.4. Dental morphospace occupation of early Palaeocene plesiadapiforms

In the two-dimensional geometric morphometric (2DGM) analysis of Lancian and early Puercan therians from northeastern Montana, archaic ungulates (plus signs) and purgatoriids (yellow stars) occupy a similar region of the morphospace, as represented by the plot of PC1 versus PC2 (figure 3*c*). That region reflects lower molars that are relatively inflated and have broad talonid basins—morphological adaptations toward omnivory and frugivory [33]. Plesiadapiforms from later in the Puercan and earliest Torrejonian (yellow, orange, pink and red stars) plot farther toward the lower-right corner of the dental morphospace, away from the early Puercan archaic ungulates and toward the region previously occupied by the latest Cretaceous frugivorous stem marsupial *Glasbius* [31,34,35].

## 3. Discussion

### 3.1. Evolutionary implications

New fossils from the Harley's Point locality document two purgatoriid taxa from the early Puercan (Pu1) of northeastern Montana, USA: *Purgatorius janisae*, previously only known from the late Puercan (Pu3) Garbani Channel localities [16], and the new taxon, *P. mckeeveri*, which we also document from the Pu3 Garbani Channel localities. These new occurrences of *Purgatorius* at the Harley's Point locality represent the oldest confirmed record of Pan-Primates and Euarchonta known. Their co-occurrence with *Protungulatum donnae*, *Mimatuta minuial* and *Thylacodon montanensis* at the Harley's Point locality [36] supports a Pu1 age. The associated, high-resolution stratigraphic and geochronological data further constrain the age of the locality to within 208 kyr post-KPB (66.052–65.844 Ma), with a likelihood that the age could be as early as 105–139 kyr post-KPB (65.946–65.912 Ma). Harley's Point locality is possibly on the younger end of this range, given that *Purgatorius* does not occur at the Pu1 Z-Line localities [28] or the well-sampled Pu1 Worm Coulee 1 locality [12,29] from *ca* 25 and 80 kyr post-KPB, respectively. The slightly younger age for Harley's Point is also consistent with the substantial thickness of its associated channel sequence (approx. 17 m) and the occurrence of a younger-aspect archaic ungulate (?periptychid, LACM 112903 [29]) from the same channel sequence (nearby McKeever Ranch 1 locality) that is unknown from those other well-sampled Pu1 localities. With few exceptions (see the electronic supplementary material), all other known occurrences of plesiadapiforms are from Pu3 (*ca* 65.6 Ma [26]) or younger [2].

A Cretaceous origin of Pan-Primates has long been hypothesized by palaeontologists, in part based on the initial description of *Purgatorius*, including *P. ceratops*, which until more recently [14,15] was considered latest Cretaceous in age [13]. A consensus later emerged that despite the lack of unambiguous records of Cretaceous plesiadapiforms, a pre-KPB origin of Pan-Primates was still within the realm of possibility (e.g. [2,16]). Fox & Scott [24] recently speculated that the early (Pu2) occurrence and derived characteristics of *Purgatorius coracis* imply that the ancestral purgatoriid was from the Late Cretaceous. Fossils reported here (i) derive from an older (Pu1) locality, less than 208 kyr and most likely to within 105–139 kyr post-KPB, and (ii) represent two sympatric species of *Purgatorius*, each with a uniquely accumulated suite of dental specializations that evolved following divergence from a common ancestor. These data provide even stronger evidence that the origin of plesiadapiforms, and in turn Pan-Primates, Euarchonta and Placentalia extends back into the Late Cretaceous.

As is the case for many other mammalian clades, there is considerable discrepancy between the fossil record and molecular-clock estimates for the timing of the origin of Primates, Euarchonta and Placentalia [37,38]. Fossil evidence is generally in accord with Placentalia and stem members of some placental orders having originated either just before or after the KPB (Soft and Hard Explosive Models, respectively) and the placental ordinal crown groups having originated and diversified in the early Cenozoic; molecular-clock studies, in contrast, support the origin of Placentalia and placental ordinal stem groups earlier in the Cretaceous and the origination and diversification of placental ordinal crown groups either shortly thereafter (Short-Fuse Model) or after the KPB (Long-Fuse Model) [39,40]. The co-occurrence of two species of *Purgatorius* documented here at *ca* 66 Ma establishes a slightly older divergence date for Pan-Primates (with Pan-Primates splitting either from Dermoptera [assuming Primatomorpha is valid] or Sundatheria [Dermoptera + Scandentia]) and for Euarchonta (with Euarchonta splitting from Glires) than previously documented in the fossil record. Minimum fossil calibrations used in recent analyses have been younger (e.g. 65 Ma for *Purgatorius* representing Euarchonta [41]; 64.85 Ma for *Purgatorius coracis* representing Euarchonta and 64.85 Ma for *Protungulatum donnae* representing Placentalia [37]) and have resulted in Pan-Primates diversifying after or just before the KPB. Although a difference of 1 Myr for a minimum fossil calibration is not necessarily regarded as significant in the context of deep time, two species of *Purgatorius* at 66 Ma might be an especially important observation given their close proximity to the KPB. The oldest date for Pan-Primates and/or Euarchonta established here provides additional support for a Cretaceous origin or a more explosive evolution of Pan-Primates in the earliest Palaeocene.

### 3.2. Palaeoecological implications

The new fossils reported here also shed light on the palaeoecology of early plesiadapiforms and, by extension, Pan-Primates and Euarchonta in relation to the post-KPB biotic recovery and the evolutionary radiation of other placental mammals. In northeastern Montana, which we use as a model for the post-KPB biotic recovery (but see also [42]), plesiadapiforms were not part of the earliest ‘disaster’ or ‘survival’ phase [12,28]. They first appear, as immigrants into the area [43], in the Harley's Point local fauna, which we estimate is slightly younger than the ‘disaster faunas’ (i.e. ‘recovery’ phase) but still within *ca* 208 kyr post-KPB (figure 3*a*, stars). We speculate that the delayed arrival and initially low relative abundance of plesiadapiforms (figure 3*b*, stars) might have been tied to their arboreal ecology [4,44,45] and the temporary loss of arboreal habitats across the KPB in the region [46]. By the ‘fully recovered’ phase (*ca* 328 to 847 kyr post-KPB), plesiadapiforms were represented by at least three *Purgatorius* spp. and *Pandemonium dis* [12,13,19] and were numerically abundant (figure 3*b* [12]). Although purgatoriids declined in relative abundance thereafter (*ca* 934 kyr to 1.01 Myr post-KPB), the paromomyid *Paromomys farrandi* made up more than half of all individuals in the local faunas of northeastern Montana [27,30].

An illuminating contrast is the corresponding pattern for archaic ungulates (figure 3*a,b*, plus signs), a group considered central to the post-KPB recovery and placental radiation [12,47,48]. They too arrived in northeastern Montana as immigrants but prior to plesiadapiforms, in the earliest ‘disaster’ phase of the post-KPB recovery (see [49,50] for possible older occurrences elsewhere). Despite their greater taxonomic richness, archaic ungulates were surpassed in numerical abundance by plesiadapiforms during the ‘fully recovered’ phase (25% versus 12%) and that gap widened markedly by 1 Myr post-KPB (59% versus 29%). Although these two taxonomic groups had distinct patterns of species richness and relative abundance, together they dominated the initial phase of the early Palaeocene placental radiation [12].

The diversification of these two groups, which continued through most of the Palaeocene, was characterized by a trend toward omnivory and herbivory [51]. Results of our 2DGM analysis of lower molars show the initiation of this dietary trend (figure 3*c*). Overlap in dental morphospace between archaic ungulates and plesiadapiforms (especially purgatoriids) should not necessarily be interpreted as direct competition for food resources between these early placental groups. Although the early Palaeocene fossil record of mammalian postcrania is sparse, the tarsals attributed to earliest Palaeocene archaic ungulates (e.g. cf. *Protungulatum*) differ from those of plesiadapiforms and other euarchontan mammals in lacking features related to arboreality [44,52]. Therefore, the immigration of *Purgatorius* probably represented the introduction of a unique arboreal mammal that had direct access to angiosperm products and associated insect pollinators that were probably not as readily available to contemporary terrestrial mammals including archaic ungulates [44].

The origin of Pan-Primates has long been thought to relate in part to a shift from a more insectivorous diet toward a more herbivorous diet [1,53,54]. This hypothesis is based in part on the observation that the earliest plesiadapiforms, such as *Purgatorius*, had lower crowned molars, rounder cusps, and broader talonid basins than those of many Late Cretaceous and contemporaneous early Palaeocene small-bodied mammals. Although many plesiadapiforms appear to have been at least partly specialized for increased consumption of non-leafy plant resources (e.g. fruit), it seems likely that all *Purgatorius* species also relied on insects to varying extents given their small body size and aspects of their molar morphology. Most previous studies on the dietary features of purgatoriid teeth have been qualitative, but a recent study [55] used dental topographic analyses to assess the diet of paromomyid plesiadapiforms and included casts of two teeth of *Purgatorius janisae* (p4, m2) and one tooth of *P. coracis* (m2) for comparative purposes. Their results suggested that *P. janisae* was an insectivore and *P. coracis* was a less strict insectivore (i.e. insectivore–omnivore), whereas the paromomyid *Paromomys farrandi* was an omnivore–frugivore [55].

Our 2DGM results are consistent with previous studies (e.g. [53,55]); purgatoriid species from northeastern Montana occupy a more insectivorous–omnivorous morphospace than larger (e.g. *Pandemonium*) and more specialized (e.g. lower crowned, more bunodont molars of *Paromomys*) plesiadapiforms that occupy more herbivorous morphospace. The two oldest known species of *Purgatorius* (this paper) evolved distinct dental specializations to capitalize on a mixed diet of insects and plant products in different ways. *Purgatorius janisae* has molars with shorter trigonids, pointed cusps and sharper crests, whereas *P*. *mckeeveri* has molars with relatively taller trigonids with more inflated cusps and rounded crests. We posit that such dental features for omnivory coupled with postcranial specializations for arboreality led to the rapid evolutionary success of the plesiadapiforms following the K/Pg mass extinction.

## Data accessibility

Associated data are available in the electronic supplementary material. All µCT tiff stacks are available via Morphosource (www.morphosource.org).
