# Supplementary material for: Earliest Palaeocene purgatoriids and the initial radiation of stem primates
Source: R Soc Open Sci. 2021 Feb 24;8(2):210050. doi: 10.1098/rsos.210050 (PMC8074693; doi:10.1098/rsos.210050)
Supplement: Supplementary file 1 [file rsos210050_review_history.pdf]

THE ROYAL SOCIETY
PUBLISHING

ROYAL SOCIETY
OPEN SCIENCE

Earliest Palaeocene purgatoriids and the initial radiation of stem primates

Gregory P. Wilson Mantilla, Stephen G. B. Chester, William A. Clemens, Jason R. Moore, Courtney J. Sprain, Brody T. Hovatter, William S. Mitchell, Wade W. Mans, Roland Mundil and Paul R. Renne

Article citation details
R. Soc. open sci. **8**: 210050.
http://dx.doi.org/10.1098/rsos.210050

Review timeline
Original submission: 13 January 2021
Final acceptance: 18 January 2021

Note: Reports are unedited and appear as submitted by the referee. The review history appears in chronological order.

Note: This manuscript was transferred from another Royal Society journal with peer review.

Review History

Decision letter (RSOS-210050.R0)

The editorial office reopened on 4 January 2021. We are working hard to catch up after the festive break. If you need advice or an extension to a deadline, please do not hesitate to let us know -- we will continue to be as flexible as possible to accommodate the changing COVID situation. We wish you a happy New Year, and hope 2021 proves to be a better year for everyone.

Dear Dr Wilson Mantilla:

It is a pleasure to accept your manuscript entitled "Earliest Palaeocene purgatoriids and the initial radiation of stem primates" in its current form for publication in Royal Society Open Science. The comments of the reviewer(s) who reviewed your manuscript are included at the foot of this letter.

You can expect to receive a proof of your article in the near future. Please contact the editorial office (openscience@royalsociety.org) and the production office (openscience_proofs@royalsociety.org) to let us know if you are likely to be away from e-mail contact -- if you are going to be away, please nominate a co-author (if available) to manage the proofing process, and ensure they are copied into your email to the journal.

Reports © 2021 The Reviewers; Decision Letters © 2021 The Reviewers and Editors; Responses © 2021 The Reviewers, Editors and Authors. Published by the Royal Society under the terms of the Creative Commons Attribution License http://creativecommons.org/licenses/by/4.0/, which permits unrestricted use, provided the original author and source are credited

Due to rapid publication and an extremely tight schedule, if comments are not received, your paper may experience a delay in publication.

Please see the Royal Society Publishing guidance on how you may share your accepted author manuscript at https://royalsociety.org/journals/ethics-policies/media-embargo/.

Thank you for your fine contribution. On behalf of the Editors of Royal Society Open Science, we look forward to your continued contributions to the Journal.

Kind regards,
Royal Society Open Science Editorial Office
Royal Society Open Science
openscience@royalsociety.org

on behalf of Professor Marcelo Sanchez (Associate Editor) and Professor Kevin Padian (Subject Editor).

Associate Editor Professor Marcelo Sanchez Comments to Author:

Associate Editor: 1
Comments to the Author:
Thank you for addressing the reviews thoughtfully and thoroughly

Follow Royal Society Publishing on Twitter: @RSocPublishing
Follow Royal Society Publishing on Facebook:
https://www.facebook.com/RoyalSocietyPublishing.FanPage/
Read Royal Society Publishing's blog:
https://royalsociety.org/blog/blogsearchpage/?category=Publishing
